# Maintenance Therapy in Ovarian Cancer with Targeted Agents Improves PFS and OS: A Systematic Review and Meta-Analysis

**DOI:** 10.1371/journal.pone.0139026

**Published:** 2015-09-24

**Authors:** Xinyu Qian, Jing Qin, Songdan Pan, Xin Li, Yuelong Pan, Shenglin Ma

**Affiliations:** 1 Department of Chemotherapy, Hangzhou First People's Hospital, Hangzhou Cancer Hospital, Hangzhou, Zhejiang, P.R. China; 2 Department of Chemotherapy, Zhejiang Cancer Hospital, Hangzhou, Zhejiang, P.R. China; Women’s Hospital, School of Medicine, Zhejiang University, China. 310006, CHINA

## Abstract

**Background:**

Maintenance therapy with targeted agents for prolonging remission for ovarian cancer patients remains controversial. As a result, a meta-analysis was conducted to assess the effectiveness and safety of using maintenance therapy with targeted agents for the treatment of ovarian cancer.

**Methods:**

From inception to January 2015, we searched for randomized, controlled trials (RCTs) using the following databases: PubMed, ScienceDirect, the Cochrane Library, Clinicaltrials.gov and EBSCO. Eligible trials included RCTs that evaluated standard chemotherapy which was either followed or not followed by targeted maintenance in patients with ovarian cancer who had been previously receiving adjunctive treatments, such as cytoreductive surgery and standard chemotherapy. The outcome measures included progression-free survival (PFS), overall survival (OS) and incidence of adverse events.

**Results:**

A total of 13 RCTs, which were published between 2006 and 2014, were found to be in accordance with our inclusion criteria. The primary meta-analysis indicated that both PFS and OS were statistically and significantly improved in the targeted maintenance therapy group as compared to the control group (PFS: HR = 0.84, 95%CI: 0.75 to 0.95, p = 0.001; OS: HR = 0.91, 95%CI: 0.84 to 0.98, p = 0.02). When taking safety into consideration, the use of targeted agents was significantly correlated with increased risks of fatigue, diarrhea, nausea, vomiting, and hypertension. However, no significant differences were found in incidence rates of abdominal pain, constipation or joint pain.

**Conclusions:**

Our results indicate that targeted maintenance therapy clearly improves the survival of ovarian cancer patients but may also increase the incidence of adverse events. Additional randomized, double-blind, placebo-controlled, multicenter investigations will be required on a larger cohort of patients to verify our findings.

## Introduction

Ovarian carcinoma is the eighth leading cause of cancer morbidity among women, the second leading cause of malignancy and the seventh leading cause of cancer-related deaths according to reports published by the WHO. Approximately 225,000 new ovarian cancer cases are diagnosed per year in women around the world, and approximately 140,000 deaths occur from ovarian carcinoma per year in women around the world [1]. Adjuvant chemotherapy following cytoreductive surgery with platinum-based treatment regimens leads to a higher response rate (approximately 75%) in ovarian carcinoma patients [2]; however, the rates of recurrence and mortality are currently still high (5-year survival rate: 20%-40%) [3]. Many previous studies have indicated that the administration of chemotherapy over a course of 6 cycles cannot improve prognosis and that it increases the risk of adverse events [4–7].

Maintenance chemotherapy is harmful to patients. So novel agents are being developed to target specific molecular pathways. Clinical data with molecular targeted agents, such as inhibitors of poly (adenosine diphosphate [ADP]-ribose), polymerase (PARP)[8], and vascular endothelial growth factor (VEGF)[9], is encouraging. There is some evidence from a meta-analysis of 4 trials that the addition of bevacizumab to standard chemotherapy may reduce the risk of disease progression, in women with advanced ovarian cancer; however, it does not improve overall survival (OS) [10]. Using bevacizumab in combination with standard chemotherapy is limited in producing a curative effect[11]. However, whether employing a consolidated treatment with targeted drugs should be further investigated. Targeted maintenance therapy is defined as maintenance administration of single-agent antibody or small-molecule inhibitor after the completion of chemotherapy.

At present, several obstetrics and gynecology researchers suggested that maintenance or consolidation treatment with targeted drugs after standard chemotherapy should be used to manage ovarian cancer patients. By doing so, this might potentially prolong remission, help overcome resistance to conventional drugs, and improve quality of life [9, 12, 13]. However, the benefits produced by targeted maintenance therapy are actually controversial. Trial by Bois [12] showed that maintenance therapy using pazopanib can improve progression-free survival (PFS) for 5 to 6 months, and Ledermann [13] reported that maintenance therapy using nintedanib also improved PFS. Conversely, many other trials (e.g., Sabbatini [14], Herzog [15]) have shown that targeted maintenance therapy is not efficacious with respect to PFS or OS and instead significantly increases the risk of adverse events compared with placebo. Some of the above discussed RCTs included a relatively small sample size, and therefore, there is no enough evidence to draw comprehensive conclusions. As a result, for patients suffering from ovarian cancer, it is critically important to balance the pros and cons of new treatments. The aim of this study was to assess and discuss the effectiveness and safety of using maintenance therapy with targeted agents in ovarian cancer patients. To accomplish this goal, a comprehensive meta-analysis was conducted to provide a reference of clinical options on the use of maintenance therapy with targeted agents to treat patients with ovarian cancer.

## Methods

### Inclusion and exclusion criteria

In this meta-analysis, only published RCTs on humans were included, and the language restriction was set to English. Trials were included if they fulfilled the following inclusion criteria: (1) patients with a definite diagnosis of ovarian cancer, and (2) who had previously received adjunctive treatments such as cytoreductive surgery and standard chemotherapy. Interventions were performed as follows: the experimental group included patients who were administered targeted maintenance therapy after standard treatment, and the control group included patients who were either treated with placebo or who were only observed following standard chemotherapy.

### Search strategy

Two reviewers (XQ and JQ) independently and simultaneously screened articles in the following databases: PubMed, ScienceDirect, Cochrane Library, Clinicaltrials.gov, and EBSCO. The retrieval time was set as from inception to January 2015. The following keywords were used to identify possible publications: “ovarian cancer,” “maintenance,” and “randomized controlled trials”. Trials that did not obviously conform to our criteria were excluded; examples of such include trials about maintenance therapy using chemotherapy, radiotherapy, or diet. Trials about maintenance therapy using targeted agents were further evaluated, and any differences that arose were resolved by a third investigator (SP).

### Quality assessment

Two members (XQ and JQ) independently completed quality assessments on the basis of the guidelines in the Cochrane Handbook for Systematic Reviews of Interventions 5.0.2 [16]. The following 7 items were extracted to assess the quality of each publication: (1) random sequence generation (selection bias), (2) allocation concealment (selection bias), (3) blinding of participants and personnel (performance bias), (4) blinding of outcome assessment (detection bias), (5) incomplete outcome data (attrition bias), (6) selective reporting (reporting bias), and (7) other bias. A third investigator (XL) was called upon to resolve any differences.

### Data extraction

The outcomes that we primarily focused on were effectiveness (PFS, OS) and safety (adverse events). The essential data was extracted from each trial in a unified format that included the first author’s name, year of publication, country in which the trial was performed, fixed number of years for patient inclusion, name of targeted agent, experimental design, cases grouped randomly, mean age and death. PFS was defined as spanning from randomization to either recurrence or death, and OS was defined as spanning from randomization to death. We extracted the hazard ratios (HRs) and 95% confidence intervals (95% CI) directly from each trial to evaluate the survival effects on PFS and OS. Tumor progression was defined according to RECIST (Response Evaluation Criteria in Solid Tumors) criteria, deterioration of overall physical condition and CA125 improvement. The number of patients that experienced adverse events was extracted directly from each trial based on safety outcomes (adverse reactions). The classification of adverse events was based on Common Terminology Criteria for Adverse Events, version 3.0.

### Statistical analysis

We comprehensively evaluated PFS, OS, and adverse events using Review Manager, version 5.2 (provided by the Cochrane Library) [17]. We then calculated OR values and corresponding 95% CIs for binary classification data and WMD, in addition to the 95% CIs corresponding to continuous variables. A fixed-effect model was used acquiescently to determine consolidation effect values. In our opinion, there was explicit heterogeneity between trials and therefore we utilized a random-effects model in cases where differences between groups (p < 0.05 and I^2^ > 60%) existed simultaneously. When I^2^ > 75%, it means an obvious statistical heterogeneity among included trials. Then sensitivity analysis was performed excluding trials at high risk of bias. We considered cases in which all of the p values that corresponded to the merger effect values (OR, HR and WMD) were lower than 0.05 to be statistically significant. The PRISMA Checklist is provided in S1 File.

## Results

### Characteristics of the included trials

**Fig 1:**
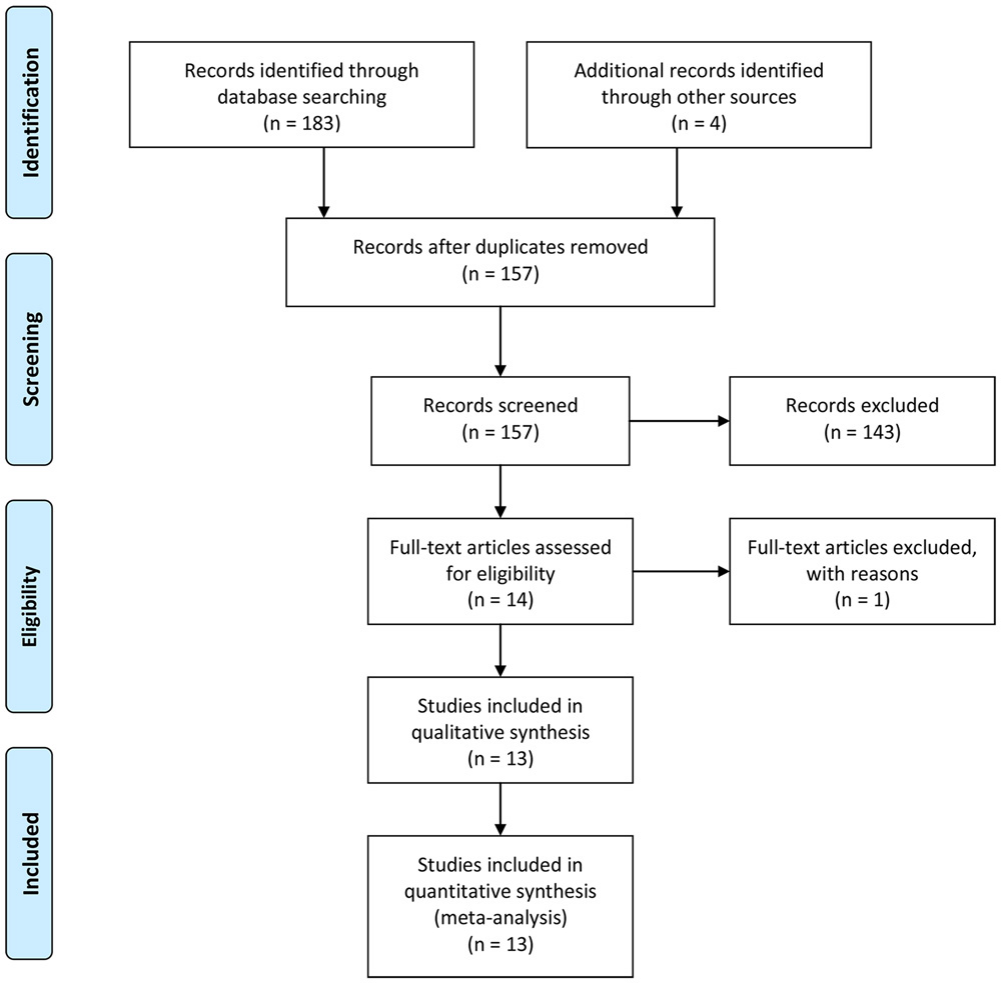
Flow diagram of trial selection.

We identified 183 papers from the above discussed databases, and 14 [8, 9, 12–15, 18–25] of these articles were included based on the search criteria, among which 1 trial [23] was excluded because the treatment group undergoing targeted maintenance therapy included only 4 patients. A final total of 13 RCTs were included in our meta-analysis. Our search strategy and the steps used to select eligible trials are summarized in the flow diagram shown in Fig 1 (for detailed information, please see S2 File). Nearly all of the patients in the included trials were initially treated with first-line chemotherapy for 6 cycles. The exceptions included the GOG-0218 trial that evaluated the use of bevacizumab in combination with standard chemotherapy as a first-line treatment in the experimental group [9] and a trial conducted by Meier in which lonafarnib was used for both first-line and maintenance therapy in the experimental group [22]. We included these two trials because of their high quality and clinical significance and also to reduce heterogeneity as much as possible. Among the included trials, 6 were phase III RCTs, and 7 were phase II RCTs. When classifying the included trials based on the mechanisms of targeted agents, 3 used monoclonal antibody, and 10 used small molecule drugs. The characteristics of the 13 included trials are summarized in Table 1.

**Table 1:** Characteristics of included trials.

Study	Year	Country	Recruitment period	Drug	RCT phase	Patients number		Age (year)		Survival outcome
						Targeted	Control	Targeted	Control	
Berek	2009	USA	2002–2007	Oregovomab	III	251	120	58.8	59.6	PFS
Bois	2014	Germany	2009–2014	Pazopanib	III	472	468	56	57	OS,PFS
Burger	2011	USA	2005–2009	Bevacizumab	III	623	625	60	60	OS,PFS
Herzog	2013	USA	2008–2009	Sorafenib	II	123	123	56.9	54.4	PFS
Hirte	2006	Canada	1998–1999	BAY 12–9566	III	122	121	58.4	56.7	OS
Karlan	2012	USA	2007–2009	AMG 386	II	53	55	59	62	OS,PFS
Kaye	2012	USA	2008–2009	Vismodegib	II	52	52	57.3	58.6	PFS
Ledermann	2011	UK	2006–2008	Nintedanib	II	43	40	58.4	61.3	OS,PFS
Ledermann	2012	UK	2008–2010	Olapanib	II	136	129	58	59	OS,PFS
Meier	2012	Germany	2006.2–2006.9	Lonafarnib	II	53	52	61	56	OS,PFS
Sabbatini	2013	USA	2006–2009	Abagovomab	III	593	295	56.3	56	OS,PFS
Vergote	2013	Belgium	2006–2012	Enzastaurin	II	69	73	53.6	54.5	PFS
Vergote	2014	Belgium	2005–2008	Erlotinib	III	420	415	59	59	OS,PFS

Abbreviations: PFS, progression-free survival; OS, overall survival.

A total of 5578 patients were included in our meta-analysis: 3010 patients were treated with maintenance therapy using targeted agents, and the remaining 2568 patients were treated with placebo (except for one patient in a control group, who was only subjected to observation after receiving standard chemotherapy) [22].

**Fig 2:**
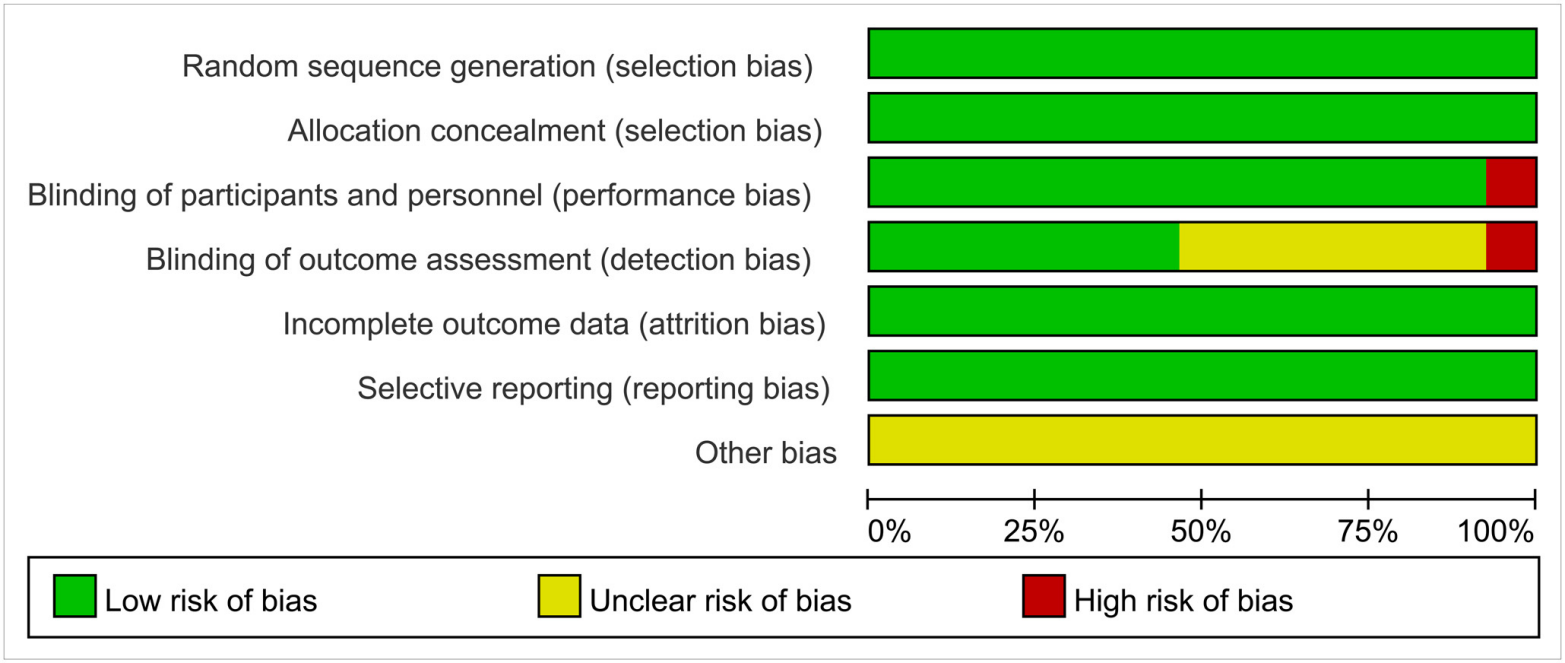
Graph of risk bias. Review of authors' judgments about each factor that was at risk of bias, which are presented as percentages across all included trials.

We presented results of the assessment of the methodological quality of each included trial in a risk of bias graph (see Fig 2) The trials conducted by Berek [18] and Sabbatini [14] performed random allocation using a centralized randomization procedure, and the trials conducted by Karlan and Ledermann [13, 20] used an automated voice response telephone system to allocate randomly; the remaining trials used stratified random allocation. Nearly all of the trials were performed in a double-blind manner, except for Meier’s trial [22]. It is additionally worth noting in total, out of all 7 trials that were included in our meta-analysis, only 150 patients received maintenance therapy with targeted agents, which is a relatively small sample size.

### Baseline comparison

The median age of the included patients before randomization was 58 years, and there were no significant differences between groups that were submitted to maintenance therapy using targeted agents versus those that were treated with placebo (WMD = -0.2, 95%CI: -0.77 to 0.37, p = 0.49). Although 84% of the patients were Caucasian, no significant differences were found between the groups submitted to maintenance therapy with targeted agents and those treated with placebo (OR = 0.95, 95%CI: 0.79 to 1.14, p = 0.55). The main histological type was serous adenocarcinoma, and the majority of tumors were stage III (FIGO stage). The life status (ECOG/WHO status) score was given priority at 0 and 1. If no significant differences existed between groups with respect to baseline information (detailed above), then we considered there to be no confounding factors in comparing safety and efficacy between the two groups. The baseline analysis details are shown in Table 2.

**Table 2:** Comparations of baseline characteristics.

Outcome or Subgroup	Studies	Patients	Statistical Method	Effect Estimate
1 Age	12	5335	WMD (IV, Fixed, 95% CI)	-0.20 [-0.77, 0.37]
2 Histology				
2.1 Serous	11	5171	OR (M-H, Fixed, 95% CI)	1.01 [0.89, 1.15]
2.2 Other	11	5171	OR (M-H, Fixed, 95% CI)	0.99 [0.87, 1.13]
3 FIGO Stage				
3.1 I-II	9	4858	OR (M-H, Fixed, 95% CI)	0.98 [0.75, 1.29]
3.2 III	9	4858	OR (M-H, Fixed, 95% CI)	0.94 [0.82, 1.08]
3.3 IV	10	4963	OR (M-H, Fixed, 95% CI)	1.07 [0.92, 1.24]
4 ECOG Status				
4.1 0	10	4443	OR (M-H, Fixed, 95% CI)	1.03 [0.91, 1.17]
4.2 1	10	4443	OR (M-H, Fixed, 95% CI)	0.99 [0.87, 1.13]
4.3 2	9	3668	OR (M-H, Fixed, 95% CI)	0.88 [0.61, 1.26]
5 Race	7	3777	OR (M-H, Fixed, 95% CI)	0.95 [0.79, 1.14]

Abbreviations: FIGO, International Federation of Gynecology and Obstetrics; ECOG, Eastern Cooperative Oncology Group; WMD, weighted mean difference; OR, odds ratio; IV, inverse variance; M-H, Mantel-haenszel.

### Effectiveness

#### Progression-free survival (PFS)

**Fig 3:**
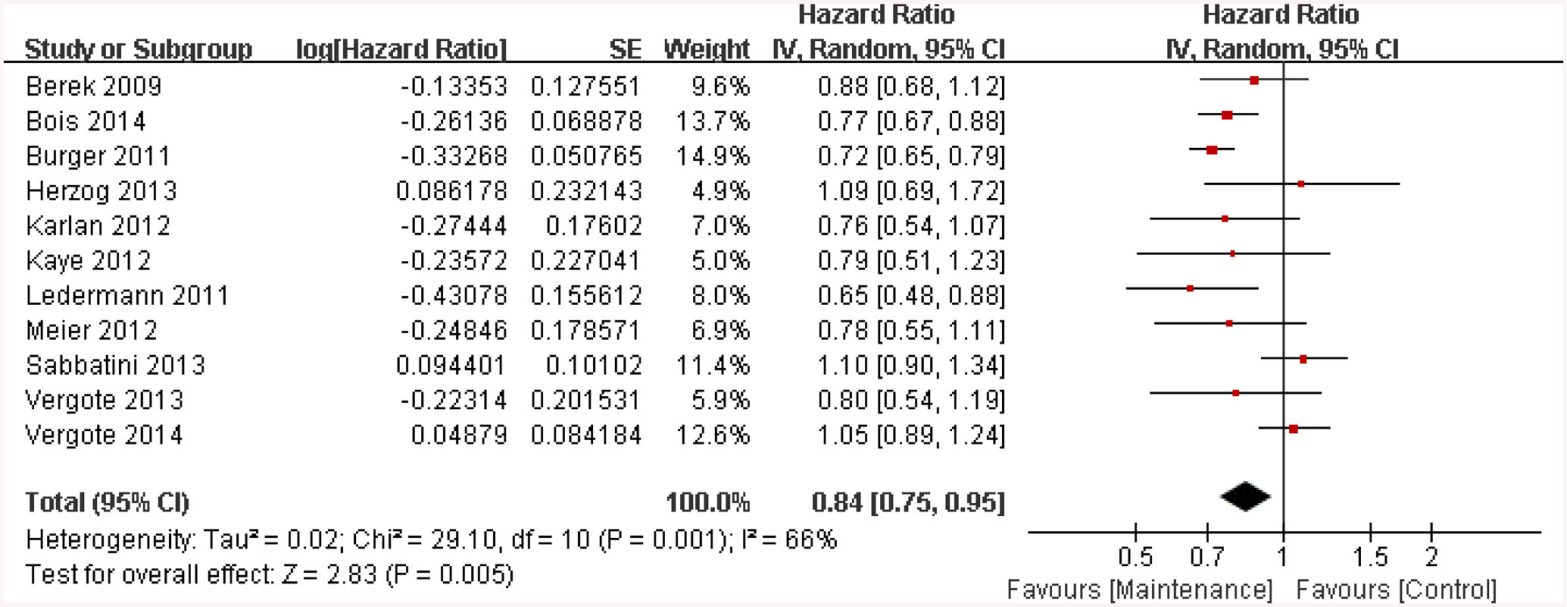
Hazard ratios of progression-free survival. SE = standard error; IV = inverse variance method; CI = confidence interval.

Almost all of the included trials (12/13) [8, 9, 12–15, 18, 20–22, 24, 25] considered PFS as the primary end point and OS or trial termination as the secondary end point. All 12 trials provided HR values regarding PFS. Here, we chose a random-effects model because of the differences that were observed between the above discussed trial groups (p < 0.0001 and I^2^ = 94%). By sensitivity analyses, we excluded the trial by Ledermann for high heterogeneity [8]. Then the heterogeneity among the left 11 trials as measured by PFS changed to an acceptable level (I^2^ = 66%). Targeted maintenance therapy was found to significantly improve PFS when compared to placebo groups (HR = 0.84, 95%CI: 0.75 to 0.95, p = 0.001, see Fig 3).

#### Overall survival (OS)

**Fig 4:**
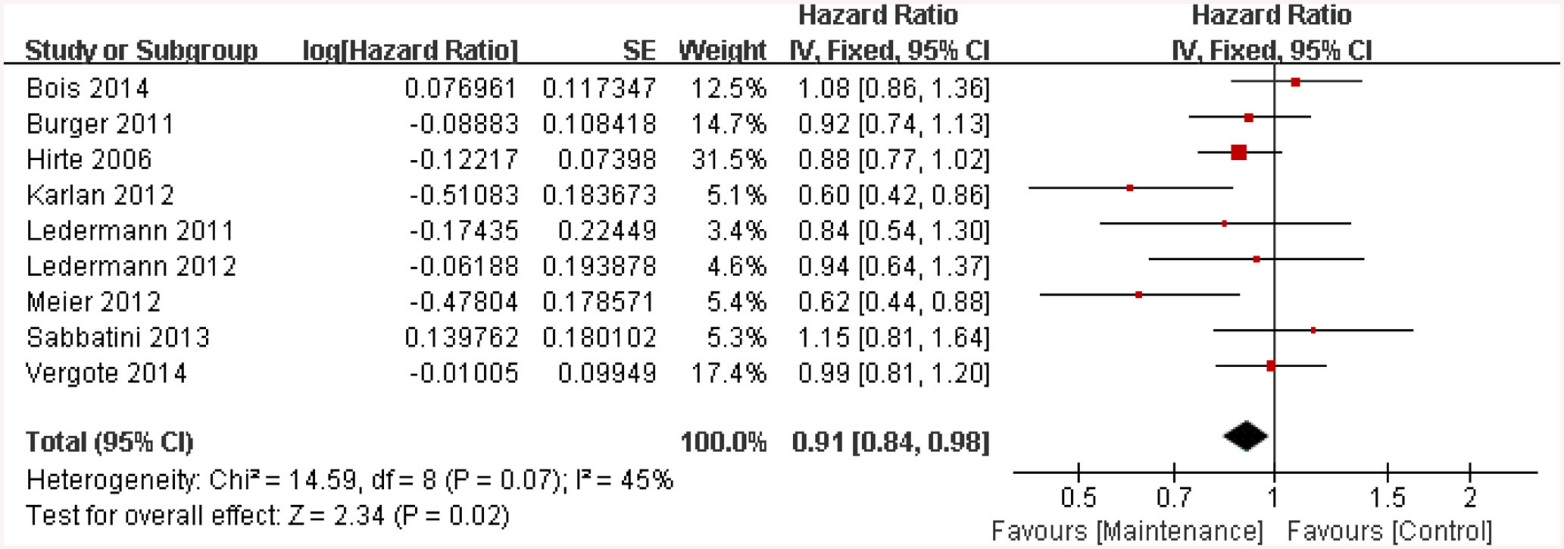
Hazard ratios of overall survival. SE = standard error; IV = inverse variance method; CI = confidence interval.

A total of 9 trials [8, 9, 12–14, 19, 20, 22, 25] provided HR values about OS. We chose a fixed-effect model to evaluate the differences between the above trials (p = 0.07 and I^2^ = 45%). Maintenance therapy with targeted agents was found to be associated with significant improvements in OS when compared to placebo groups (HR = 0.91, 95%CI: 0.84 to 0.98, p = 0.02, see Fig 4).

### Safety (adverse events)

**Fig 5:**
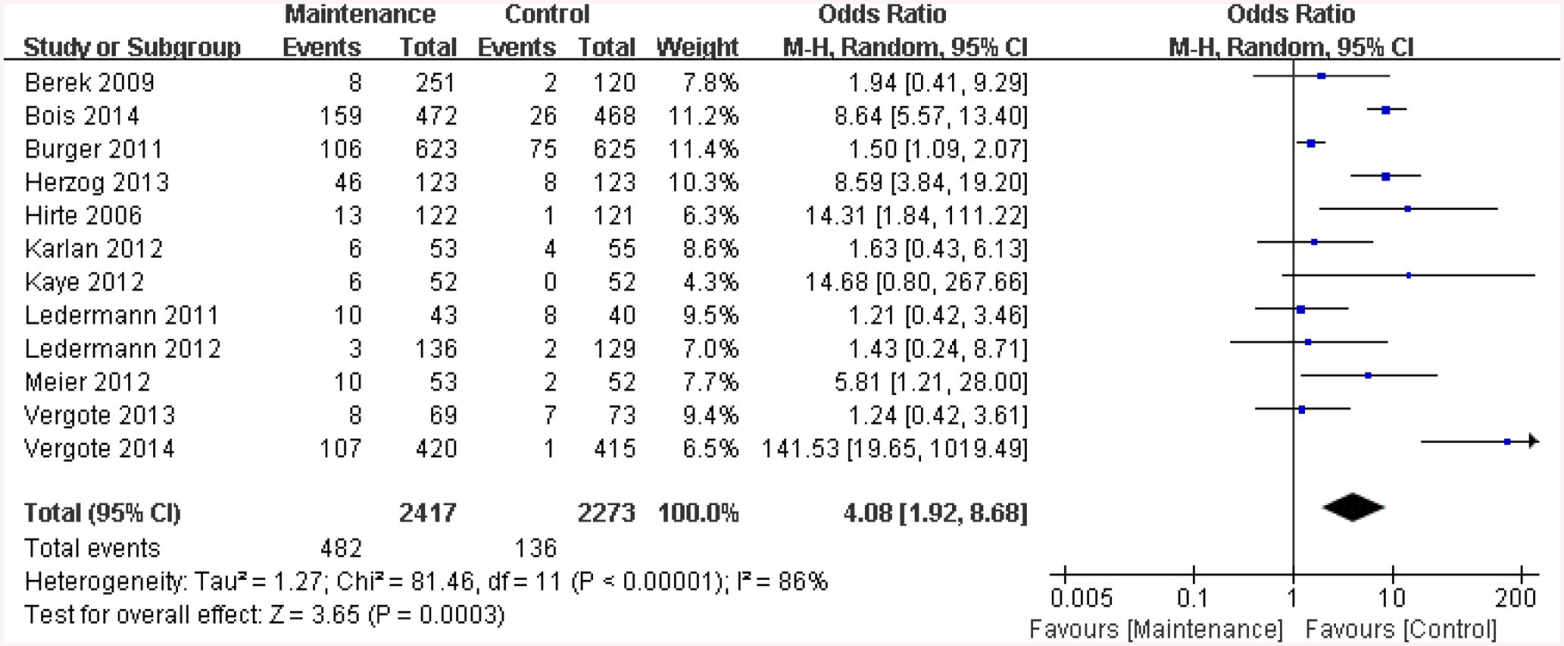
Risk of discontinuing treatment due to adverse events (CTCAE≥3) in the targeted maintenance therapy group versus the control group. M-H = Mantel-Haenszel method; CI = confidence interval. CTCAE: Common Terminology Criteria for Adverse Events.

Generally, level 3/4 grade adverse events (according to CTCAE: Common Terminology Criteria for Adverse Events) are considered to affect quality of life [18, 22]. Therefore, we assessed the quality of life in the patients that experienced level 3/4 adverse events. As shown in Table 3, adverse events that were reported in more than 5 of the trials were defined as abdominal pain, fatigue, diarrhea, nausea, constipation, vomiting, hypertension, and joint pain. Pool OR values suggested that maintenance therapy using targeted agents significantly increased the incidence of fatigue (OR = 2.72, 95%CI: 1.44 to 13, p = 0.002), diarrhea (OR = 4.77, 95%CI: 2.68 to 8.48, p < 0.001), nausea (OR = 3.63, 95%CI: 1.09 to 12.03, p = 0.04), vomiting (OR = 2.86, 95%CI: 1.07 to 7.68, p = 0.04), hypertension (OR = 4.44, 95%CI: 3.16 to 6.22, p < 0.001) but did not markedly increase the incidence of abdominal pain (OR = 1.10, 95%CI: 0.69 to 1.76, p = 0.42), constipation (OR = 0.69, 95%CI: 0.22 to 2.15, p = 0.53) or joint pain (OR = 0.97, 95%CI: 0.30 to 3.18, p = 0.96). Overall, the risk of withdrawal of treatment as a result of adverse events was significantly increased in the targeted maintenance therapy groups when compared to the placebo groups (OR = 4.08, 95%CI: 1.92 to 8.68, p < 0.001 and I^2^ = 86%; see Fig 5). The obvious heterogeneity might be related to the prevention of risks, benefits, and financial costs. The conclusion assessing withdrawal of treatment should be used cautiously.

**Table 3:** Adverse events.

Outcome or Subgroup	Studies	Patients	Statistical Method	Effect Estimate
1 Abdominal pain	8	3063	OR (M-H, Fixed, 95% CI)	1.10 [0.69, 1.76]
2 Fatigue	7	1981	OR (M-H, Fixed, 95% CI)	2.72 [1.44, 13]
3 Diarrhea	7	1981	OR (M-H, Fixed, 95% CI)	4.77 [2.68, 8.48]
4 Nausea	6	1043	OR (M-H, Fixed, 95% CI)	3.63 [1.09, 12.03]
5 Constipation	5	803	OR (M-H, Fixed, 95% CI)	0.69 [0.22, 2.15]
6 Vomiting	5	803	OR (M-H, Fixed, 95% CI)	2.86 [1.07, 7.68]
7 Hypertension	5	2583	OR (M-H, Fixed, 95% CI)	4.44 [3.16, 6.22]
8 Joint pain	5	1658	OR (M-H, Fixed, 95% CI)	0.97 [0.30, 3.18]

Abbreviations: OR, odds ratio; M-H, Mantel-haenszel.

### Subgroup analysis

Two subgroups were initially divided based on whether targeted agents were combined with first-line therapy. In 2 trials, standard chemotherapy was combined with targeted agents as the first-line therapy, whereas in the remaining 11 trials the first-line therapy was standard chemotherapy only. There were no significant differences in therapeutic effectiveness (PFS, OS) between subgroups. There were also no significant differences between subgroups according to which targeted agents were employed (monoclonal antibody vs. small molecules). Finally, 2 subgroups were divided according to the withdrawal rate (>30% vs. <30%) of targeted maintenance therapy, and the results indicated that there were significant differences among them. One of the subgroups was insufficiently studied, which reduces the credibility of the results that were obtained in the above subgroup analyses.

## Discussion

In performing a comprehensive analysis of 13 RCTs that were focused on targeted maintenance therapy, we found that maintenance therapy using targeted agents led to significant improvements in both PFS and OS in ovarian cancer patients compared to maintenance therapy using placebo. However, targeted maintenance therapy was also found to increase the occurrence of adverse events such as fatigue, diarrhea, nausea, vomiting, and hypertension.

It has been previously shown that targeted maintenance therapy leads to a better prognosis for patients with malignancies such as non-small cell lung cancer [26] and colorectal cancer [27]. To date, ovarian cancer has consistently ranked first in mortality among gynecological tumors, and thus an increasing number of RCTs are focusing on using maintenance therapy with targeted agents to treat patients suffering from ovarian cancer.

Almost half of the trials that were included in our meta-analysis were high-quality phase III RCTs. Furthermore, the majority of them were conducted in the United States, and the remaining were conducted in various European countries. Many of the trials included in our analysis were conducted by representative multi-center tumor cooperative groups, such as the Gynecologic Oncology Group, the Canada Clinical Trials Group Study, and the European Organization for Research and Treatment of Cancer-Gynecological Cancer Group (EORTC-GCG). However, more than half of the included trials had relatively small sample sizes with respect to the targeted maintenance therapy groups. As inherent limitations and inconsistent conclusions were found among previous trials, it was therefore necessary for us to synthesize as many trials as possible to obtain the most credible evidence.

The articles included in our analysis were published between 2006 and 2014; thus, they provided relatively new data. Additionally, first-line therapy treatment regimens did not substantially change over this period of time, and worldwide measures of PFS and OS in ovarian cancer patients also did not significantly improve [28, 29]. Furthermore, in the included trials, there were no significant differences in the baseline information of patients being submitted to maintenance therapy with targeted agents compared to those being submitted to treatment with placebo, which made this meta-analysis more relevant.

In analyzing the efficacy of maintenance therapy with targeted agents compared to placebo, we found that targeted agents can significantly improve PFS and OS. However, the mechanism that is driving these findings remains undefined. Based on our safety analysis, the most common adverse events that were found to affect quality of life included fatigue, diarrhea, nausea, vomiting, and hypertension. It must be noted that the number of patients who stopped taking targeted agents due to serious adverse events was four times more than the number of patients who stopped taking placebo. Furthermore, the drug dosages initially employed had to be reduced as a result of the occurrence adverse events. This suggested that intensive monitoring of adverse events should be performed and proper interventions should be applied at the initiation of therapy, or even before therapy begins. To date, the use of maintenance therapy with targeted agents has not been popular in clinical practice because it requires adaptations and dosage adjustments to standard treatment regimens and incurs the need to prevent and treat adverse reactions.

The main advantages of our meta-analysis were as follows: a novel controversy was focused on, phase II and III RCTs were included concurrently, and a low risk of bias was indicated by Cochrane bias risk assessment. Additionally, our baseline comparison indicated that there was no obvious inclusion bias. Furthermore, we accounted for both safety and efficacy, balancing their pros and cons. Finally, our conclusions were relatively reliable and comprehensive.

Certainly, some limitations also existed in our analysis. First, the proportion of Caucasians among all trial participants was nearly 84%. Because the morbidity of ovarian cancer varies with respect to different races [30], there is no explicit answer as to whether there are differences in the curative effects of targeted maintenance therapy among different races of ovarian cancer patients. Thus, future trials are necessary to determine whether the conclusions of our meta-analysis conclusions can be applied to other races. Second, the trials we examined included a variety of targeted agents for maintenance therapy. As a result, we were unable to determine which type of agent is the most suitable for a given patient. Additionally, the high incidence of adverse events led to interruptions in maintenance therapy with targeted agents in several patients, which may have diluted our safety and efficacy results. To improve the quality of life in ovarian cancer patients, we must regularly monitor and prevent adverse events. Third, obvious statistical heterogeneity among included trials made some conclusions cautiously. We would update this meta-analysis once results of the several ongoing trials were available. Finally, the ideal timing and duration of maintenance therapies using different targeted agents requires further investigation, although the latest meta-analyses demonstrated that using bevacizumab during and after first-line therapy showed improvement in PFS but not in OS [31, 32].

In conclusion, the results of our meta-analysis suggested that maintenance therapy with targeted agents may not only postpone the progress of ovarian cancer but may also improve survival. However, this treatment approach also increases the incidence of adverse events that are related to ovarian cancer. In our opinion, the above discussed pros and cons must be weighed with respect to the clinical application of using targeted maintenance therapy in ovarian cancer patients. Additional multi-center RCTs with larger patient cohorts should be required before maintenance therapy with targeted agents becomes a widely used clinical choice for the treatment of ovarian cancer.

## Supporting Information

S1 File PRISMA Checklist. (DOC)

S2 File Search strategy and trial selection. (DOCX)
