# Supplementary material for: Maintenance Therapy in Ovarian Cancer with Targeted Agents Improves PFS and OS: A Systematic Review and Meta-Analysis
Source: PLoS One. 2015 Sep 24;10(9):e0139026. doi: 10.1371/journal.pone.0139026 (PMC4581706; doi:10.1371/journal.pone.0139026)
Supplement: S2 File — (DOCX) [file pone.0139026.s002.docx]

Search Strategy

PubMed

From inception to January 2015

1. maintenance[Title/Abstract]
2. "ovarian neoplasms"[MeSH Terms]
3. survival[Title/Abstract]
4. "random allocation"[MeSH Terms]
5. "random"[All Fields] AND "allocation"[All Fields]
6. "random allocation"[All Fields]
7. "random"[All Fields]
8. #4 or #5 or #6 or #7
9. "randomized"[All Fields]
10. #4 or #5 or #6 or #9
11. #8 or #10
12. #1 and #2 and #3 and #11

ScienceDirect

From inception to January 2015

TITLE-ABSTR-KEY(ovarian cancer) and TITLE-ABSTR-KEY(maintenance)[All Sources(Medicine and Dentistry)]

Cochrane Library

From inception to January 2015

1. ovarian cancer
2. maintenance
3. random
4. #1 and #2 and #3
5. #4 in Trials

Clinicaltrials.gov

From inception to January 2015

"ovarian cancer" AND "maintenance"

EBSCO

From inception to January 2015

1. AB ovarian cancer
2. AB maintenance
3. TX random
4. #1 and #2 and #3 and #4

Key: AB = abstract; TX = all text.


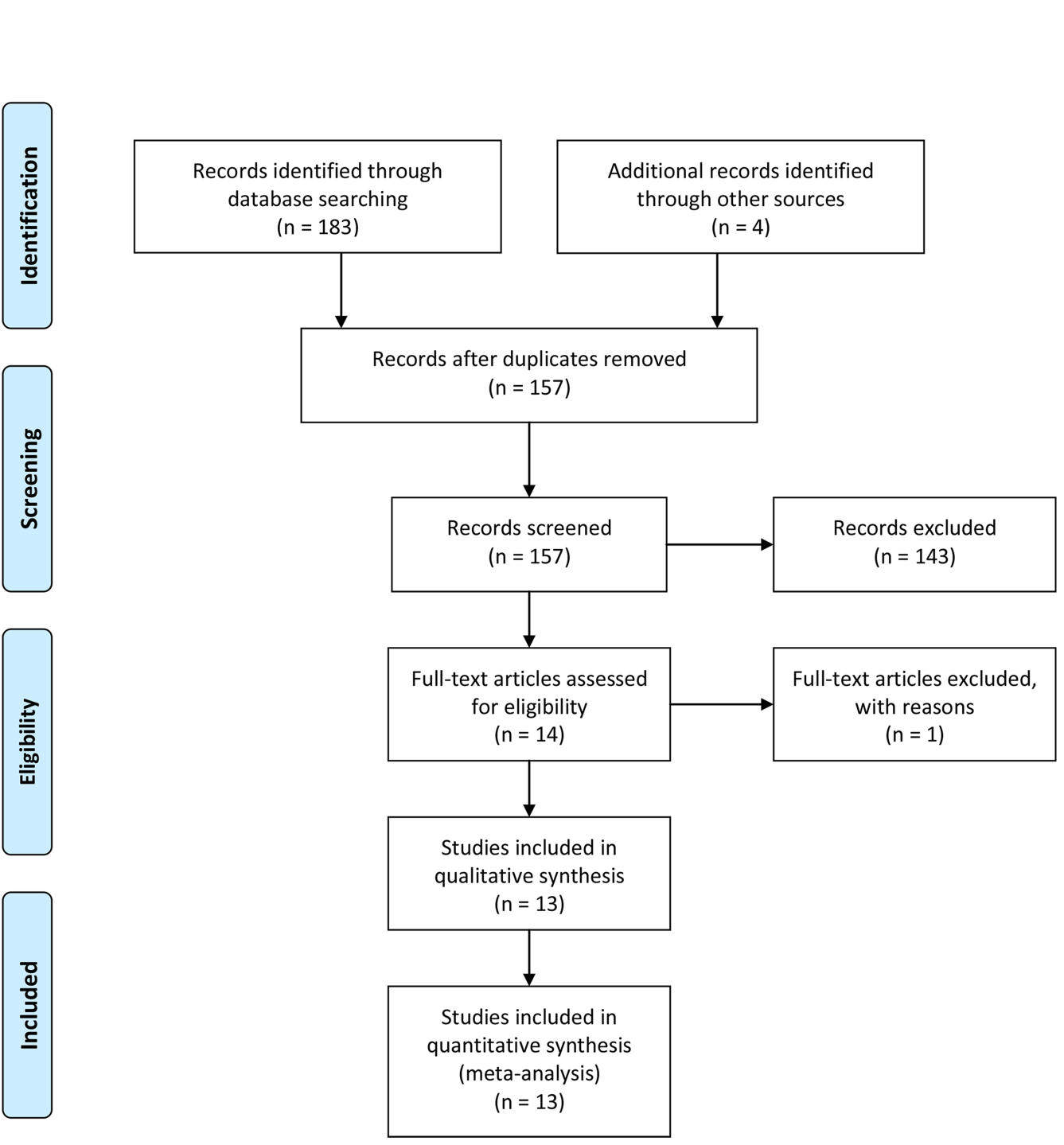


**Fig. 1** Flow diagram of trial selection

Records identified through database searching (n = 183)

Additional records identified through other sources (n = 4) [[1-4](#_ENREF_1)]

Full-text articles assessed for eligibility (n = 14) [[1](#_ENREF_1), [3](#_ENREF_3), [5-16](#_ENREF_5)]

Full-text articles excluded, with reasons (n = 1) [[13](#_ENREF_13)]

Reason: the treatment group undergoing targeted maintenance therapy included only 4 patients.

Finally, RCTs included in this meta-analysis (n = 13) [[1](#_ENREF_1), [3](#_ENREF_3), [5-12](#_ENREF_5), [14-16](#_ENREF_14)]

1. Burger RA, Brady MF, Bookman MA, Fleming GF, Monk BJ, Huang H, et al. Incorporation of bevacizumab in the primary treatment of ovarian cancer. The New England journal of medicine. 2011;365(26):2473-83. Epub 2011/12/30. doi: 10.1056/NEJMoa1104390. PubMed PMID: 22204724.

2. Galsky MD, Von Hoff DD, Neubauer M, Anderson T, Fleming M, Nagarwala Y, et al. Target-specific, histology-independent, randomized discontinuation study of lapatinib in patients with HER2-amplified solid tumors. Investigational new drugs. 2012;30(2):695-701. Epub 2010/09/22. doi: 10.1007/s10637-010-9541-0. PubMed PMID: 20857170.

3. Karlan BY, Oza AM, Richardson GE, Provencher DM, Hansen VL, Buck M, et al. Randomized, double-blind, placebo-controlled phase II study of AMG 386 combined with weekly paclitaxel in patients with recurrent ovarian cancer. Journal of clinical oncology : official journal of the American Society of Clinical Oncology. 2012;30(4):362-71. Epub 2011/12/21. doi: 10.1200/jco.2010.34.3178. PubMed PMID: 22184370.

4. Konstantinopoulos PA, Berlin ST, Campos SM, Matulonis UA, Cannistra SA. Bevacizumab rechallenge after first line maintenance bevacizumab. Gynecologic oncology. 2012;125(2):510-1. Epub 2012/03/01. doi: 10.1016/j.ygyno.2012.02.013. PubMed PMID: 22366591.

5. Berek J, Taylor P, McGuire W, Smith LM, Schultes B, Nicodemus CF. Oregovomab maintenance monoimmunotherapy does not improve outcomes in advanced ovarian cancer. Journal of clinical oncology : official journal of the American Society of Clinical Oncology. 2009;27(3):418-25. Epub 2008/12/17. doi: 10.1200/jco.2008.17.8400. PubMed PMID: 19075271.

6. du Bois A, Floquet A, Kim J-W, Rau J, del Campo JM, Friedlander M, et al. Incorporation of pazopanib in maintenance therapy of ovarian cancer. Journal Of Clinical Oncology: Official Journal Of The American Society Of Clinical Oncology. 2014;32(30):3374-82. doi: 10.1200/JCO.2014.55.7348. PubMed PMID: 25225436.

7. Herzog TJ, Scambia G, Kim BG, Lhomme C, Markowska J, Ray-Coquard I, et al. A randomized phase II trial of maintenance therapy with Sorafenib in front-line ovarian carcinoma. Gynecologic oncology. 2013;130(1):25-30. Epub 2013/04/18. doi: 10.1016/j.ygyno.2013.04.011. PubMed PMID: 23591401.

8. Hirte H, Vergote IB, Jeffrey JR, Grimshaw RN, Coppieters S, Schwartz B, et al. A phase III randomized trial of BAY 12-9566 (tanomastat) as maintenance therapy in patients with advanced ovarian cancer responsive to primary surgery and paclitaxel/platinum containing chemotherapy: a National Cancer Institute of Canada Clinical Trials Group Study. Gynecologic oncology. 2006;102(2):300-8. Epub 2006/01/31. doi: 10.1016/j.ygyno.2005.12.020. PubMed PMID: 16442153.

9. Kaye SB, Fehrenbacher L, Holloway R, Amit A, Karlan B, Slomovitz B, et al. A phase II, randomized, placebo-controlled study of vismodegib as maintenance therapy in patients with ovarian cancer in second or third complete remission. Clinical cancer research : an official journal of the American Association for Cancer Research. 2012;18(23):6509-18. Epub 2012/10/04. doi: 10.1158/1078-0432.ccr-12-1796. PubMed PMID: 23032746.

10. Ledermann J, Harter P, Gourley C, Friedlander M, Vergote I, Rustin G, et al. Olaparib maintenance therapy in platinum-sensitive relapsed ovarian cancer. The New England journal of medicine. 2012;366(15):1382-92. Epub 2012/03/29. doi: 10.1056/NEJMoa1105535. PubMed PMID: 22452356.

11. Ledermann JA, Hackshaw A, Kaye S, Jayson G, Gabra H, McNeish I, et al. Randomized phase II placebo-controlled trial of maintenance therapy using the oral triple angiokinase inhibitor BIBF 1120 after chemotherapy for relapsed ovarian cancer. Journal of clinical oncology : official journal of the American Society of Clinical Oncology. 2011;29(28):3798-804. Epub 2011/08/24. doi: 10.1200/jco.2010.33.5208. PubMed PMID: 21859991.

12. Meier W, du Bois A, Rau J, Gropp-Meier M, Baumann K, Huober J, et al. Randomized phase II trial of carboplatin and paclitaxel with or without lonafarnib in first-line treatment of epithelial ovarian cancer stage IIB-IV. Gynecologic oncology. 2012;126(2):236-40. Epub 2012/05/09. doi: 10.1016/j.ygyno.2012.04.050. PubMed PMID: 22564713.

13. Polcher M, Eckhardt M, Coch C, Wolfgarten M, Kubler K, Hartmann G, et al. Sorafenib in combination with carboplatin and paclitaxel as neoadjuvant chemotherapy in patients with advanced ovarian cancer. Cancer chemotherapy and pharmacology. 2010;66(1):203-7. Epub 2010/03/06. doi: 10.1007/s00280-010-1276-2. PubMed PMID: 20204367.

14. Sabbatini P, Harter P, Scambia G, Sehouli J, Meier W, Wimberger P, et al. Abagovomab as maintenance therapy in patients with epithelial ovarian cancer: a phase III trial of the AGO OVAR, COGI, GINECO, and GEICO--the MIMOSA study. Journal of clinical oncology [Internet]. 2013; 31(12):[1554-61 pp.]. Available from: <http://onlinelibrary.wiley.com/o/cochrane/clcentral/articles/963/CN-00964963/frame.html>.

15. Vergote IB, Chekerov R, Amant F, Harter P, Casado A, Emerich J, et al. Randomized, phase II, placebo-controlled, double-blind study with and without enzastaurin in combination with paclitaxel and carboplatin as first-line treatment followed by maintenance treatment in advanced ovarian cancer. Journal of clinical oncology : official journal of the American Society of Clinical Oncology. 2013;31(25):3127-32. Epub 2013/07/31. doi: 10.1200/jco.2012.44.9116. PubMed PMID: 23897968.

16. Vergote IB, Jimeno A, Joly F, Katsaros D, Coens C, Despierre E, et al. Randomized phase III study of erlotinib versus observation in patients with no evidence of disease progression after first-line platin-based chemotherapy for ovarian carcinoma: a European Organisation for Research and Treatment of Cancer-Gynaecological Cancer Group, and Gynecologic Cancer Intergroup study. Journal of clinical oncology : official journal of the American Society of Clinical Oncology. 2014;32(4):320-6. Epub 2013/12/25. doi: 10.1200/jco.2013.50.5669. PubMed PMID: 24366937.
